# Assessing the Satisfaction and Usability of Patient Families in the ICU With the Use of the Telehealth Communication Application myVisit

**DOI:** 10.7759/cureus.38078

**Published:** 2023-04-24

**Authors:** Thamer M Aledreesi, Meshal Alrewaished

**Affiliations:** 1 Health Informatics, King Saud Bin Abdulaziz University for Health Sciences, Riyadh, SAU; 2 Health Informatics, King Abdullah International Medical Research Center, Riyadh, SAU; 3 Health Informatics, Ministry of National Guard Health Affairs, Riyadh, SAU

**Keywords:** family support, coronavirus, intensive care unit, telehealth, myvisit application

## Abstract

Background: During the Covid-19 pandemic, there were many restrictions on family meetings, especially on patients' families meeting their dear ones in hospitals. We aimed to evaluate patients' family members' experience using the mobile application myVisit which was developed in KAMC, to connect patients in the ICU to their families and allow them to talk to them securely.

Methodology: We conducted a cross-sectional study with mixed qualitative and quantitative methods, using the technique of thematic analysis for a qualitative approach to assess user satisfaction responses and a quantitative approach to using a validated survey and comparing our findings in both methods to determine existing usability issues and potential improvements. The survey included two sections, closed and open-ended questions, which were distributed online to 63 patient family members.

Results: The response rate was 85%, the overall mean score for the first part of the closed questions (advantages of using myVisit telehealth) was 4.32 and for the second part of the scale on the ease of use of the system (advantages of using myVisit telehealth) was 3.52. Three useful topics were generated concerning the open questions, covering 220 codes from the participants' responses. In general, there is a great interest in technology and its ability to improve people's lives, especially in the medical field and cases where things do not go normally, as well as in exceptional circumstances.

Conclusions: The overall evaluation of the myVisit application was positive in terms of the idea and content, as usability of the system is very good at 71%, plus the users' opinions of the myVisit application are that it saves time at 96%, and save money and effort for the patient's family 74%.

## Introduction

The Covid-19 pandemic is a global health crisis, resulting in a significant shortage of personal protective equipment, mechanical ventilators, and intensive care unit (ICU) beds. Although emergency departments, as the primary entry point for patients having symptoms of the disease, had to adapt quickly to prioritize the safety of patients and healthcare providers. At the same time, continuing to provide the required healthcare, healthcare institutions faced significant challenges in this period, such as communicating with patients isolated in isolation departments or the ICU and enabling them to communicate with their families [1].

The presence of the family around the patient and their support is very important during crises. However, family care for their patients may be threatened during crises, as most of the time, the essence of health care is cooperation between the family and health care providers. However, maintaining public health safety is necessary in some cases. Sometimes the presence of the patient's families in hospitals is restricted, so health systems must establish procedures and adopt tools that bypass the restrictions imposed on families to visit their patients [2].

During the coronavirus pandemic, strict restrictions were imposed on visitors to visit patients inside the hospital; this strange case affected the patient and his family [1]. Also, the effect of this on the medical decision-making process, especially for patients who cannot make a decision, especially while their families are naturally not there with them to help them do so, leads to asking healthcare providers to decide for them [3]. Families’ lack of visits to their patients and their lack of participation in their care, especially when the attending physician, needs to share the patient’s health condition sometimes leads to the deterioration of the patient’s health condition [4].

One of the essential benefits of families supporting their patients in ICU is the contribution of telehealth (TH) applications to improving the communication mechanism between patients and families on the one hand and medical care providers on the other [5]. The use and continuous expansion of TH applications have helped healthcare institutions provide healthcare during disease outbreaks, especially during coronavirus, and apply the concept of social distancing [6].

Using TH services by patients themselves while they are at home to contact the treating doctor, the patient feels that he is receiving care while he is safely in his home, knowing that this service may not be a substitute for a personal visit to the doctor. However, it provides comfort because of the distance from the possibility of disease [6]. Many obstacles face medical care institutions around the world during the rapid implementation of TH services, such as slow internet connection, lack of equipment, older adults, and difficulties related to general technology, all of which led to slowing down the implementation of TH services remotely. However, medical care institutions exceeded these obstacles through the use of diverse applications, patient education, and provider/patient flexibility. Among the obstacles are also not being accepted by health care providers themselves, as older doctors face a significant challenge with technology. However, they can be educated and trained on this technology [6].

In our project, we assessed the use of the myVisit application would achieve the general objective of identifying the extent of patients' families' satisfaction with their use of the myVisit application to communicate with their patients in the ICU at the National Guard Health Affairs (NGHA) in Riyadh, through their answers to a questionnaire prepared in advance for this purpose.

The term TH appeared in the early sixties and continued to develop with the advancement of modern technologies for patient care. TH can take many forms, starting with a phone call between the doctor and the patient about his health condition or more advanced such as sharing digital patient information and using smartphone technologies.

The use of TH applications is widespread in the twenty-first century that focuses on the patient and protects doctors and patients, as TH services applications are the process of providing healthcare services by healthcare providers with the application of the concept of social distancing through the use of information and communication technology.

The study showed the importance of using healthcare applications and its positive impact on healthcare services, and the importance of providing remote healthcare is that it is useful because of the reduction in hospital admission, especially for individuals who have difficulty moving, far from their geographical locations, or who unable to access health services [1,7]. Also, the use of TH applications helps health systems in monitoring and diagnosing individuals with viral diseases such as coronavirus, thus controlling the spread of infection. TH applications also have the ability to integrate many health service providers and patient cases into one virtual network, as this network can contain central and remote clinics, private clinics, doctors' offices, rehabilitation centers, and patient records, which reduces the unification of people in one place and will reduce from the possibility of transmitting diseases [8]. TH applications have become a basic need for people and healthcare providers, as well as for patients with infectious diseases, and those in quarantine. In brief, TH applications are mainly used in crises to reduce disease transmission, improve patients' health status, ensure the safety of health care providers to provide health services, and finally, relieve pressure on health care providers and health service providers [9].

TH applications play an essential role remotely in spreading awareness messages about public health and controlling the spread of diseases, especially coronavirus disease. Many TH applications are available to facilitate the provision of health care to people with coronavirus and track patients and their contacts. The Ministry of Health in the Kingdom of Saudi Arabia has introduced additional features for TH applications services in order to be used effectively by patients and from these applications (Sehaty, Tawakklna, Tabaud, Mawid, and Tetamman) as the presence of these TH applications was necessary to control the spread of the epidemic [10]. Those applications also helped reduce infections in the Kingdom, where TH applications have facilitated the monitoring of chronic cases and reduced visits to hospitals, especially for children and the elderly at risk of disease. It also helped avoid common hospital infections [11]. The Ministry of Health encouraged people in the Kingdom of Saudi Arabia to use TH applications instead of visiting clinics and hospitals during disease outbreaks, as TH applications play an important role in primary health services to help reduce the spread of diseases, especially coronavirus [8].

## Materials and methods

We conducted a mixed-methods cross-sectional study where we collected quantitative and qualitative data to assess the usability and user satisfaction with the myVisit application. For the quantitative approach, we used the System Usability Scale (SUS) to evaluate the usability of the myVisit app. As for the qualitative approach, the thematic analysis technique was used to assess user satisfaction with the application.

We have used an online survey for data collection, consisting of demographic, open-ended, and closed-ended questions using a 5-point Likert scale. A questionnaire was written in Arabic and distributed in King Abdulaziz Medical City, National Guard-Health Affairs ICU in Riyadh, Saudi Arabia. The research community consisted of all families allowed to use myVisit to communicate with their patients in ICU. The nursing department of the ICU distributed the questionnaire by sending a barcode printed on the user manual of the myVisit application, where the participant is asked to fill out the questionnaire after the virtual visit between the patient and his family.

In this study, we used the convenience sample technique to facilitate access and contact with the target sample. At the beginning of the preparation of the study, the goal was to evaluate the responses of 25 families using the myVisit application as a total sample size; the questionnaire was distributed to 63 individuals who were family members of the patient, 42 individuals were subsequently contacted by telephone to ensure that the questionnaire was completed, and two individuals were also interviewed in person to complete the questionnaire.

The inclusion criteria included all family patients, their patients admitted to the ICU, and adults over 18 years old, also they should be fluent in reading and writing in Arabic and are eligible to use the myVisit application; on the other hand, the exclusion criteria cover all family patients less than 18 years old, and not fluent in reading and writing in Arabic, also not eligible to use the myVisit application.

We analyzed the methods of Braun and Clarke for qualitative data [10]. Then we used the SUS to assess usability by analyzing quantitative data collected from the survey [12].

## Results

Demographic

The questionnaire was distributed to 63 members of the patient's family, 54 people filled out the questionnaire and all were accepted, of 63 who consented to participate, nine failed to respond to the invitation, and the response rate of the number of people who received and filled out the questionnaire is 85%.

Below is a table showing the distribution of the sample, percentages, and frequency according to the answers to the questionnaire. Table 1 shows the distribution of the sample members according to the variables of gender, age, and educational level.

**Table 1:** Distribution of the total study sample according to demographic characteristics.

Gender	Frequency
Female	24
Male	30
Total	54
Age	Frequency
18 to 34	20
34 to 44	18
44 to 54	7
54 to 64	2
More than 64	7
Total	54
Educational level	Frequency
Bachelor	21
Diploma	8
High school and less	20
Master	4
PhD	1
Total	54

Data coding

The responses were categorized into challenges, benefits, expectations, and suggestions according to the four sections of the questionnaire. Eight codes were acquired from the participant's responses to four different questions. Table 2 below summarizes all the themes that were mentioned in the survey and shows the percentages for each theme in each category.

**Table 2:** Coding of responses in each category and repetitions of each code.

Category	Repetitions	Percent
Challenges section		
Application use	35	61%
Time	16	28%
Benefit section		
Medical	33	66%
Evaluation	10	20%
Expectation section		
Evaluation	24	50%
Medical	16	33%
Suggestion section		
Communications	16	48%
Time	6	18%

Theme-based analysis: Our analysis of the codes yielded two main themes

Theme 1

Potentially useful with training and better availability: Sixty percent of the participants mentioned difficulty using the application on both sides. Using the application involved help from the ICU staff the participant "expected communication to be direct and without prior coordination". Also, the relatives expected the application to be available 24/7 and allow more time for communication time, which was not the case "would like to increase the time of the call".

Theme 2

Keeping in touch safely: The majority of the participants mentioned praised the application for being a convenient method to see their relatives without the risk of infection transmission. The relatives cared more about how better communication with the patients helps improve the patient's motivation to recover and improve their health outcomes. Other participants mentioned that the application helps control the relative's anxiety and reduce their concern because it helps "tracking the patient's health condition and reducing the number of visits to the doctor".

Close-ended questions and quantitative analysis

The means and standard deviations were extracted to identify the responses of the members of the sample community participating in the questionnaire about assessing the satisfaction and usability of patient-families in the ICU with the use of the TH communication myVisit application and the following were the results for the benefits of adopting myVisit TH application section (Table 3).

**Table 3:** Means and standard deviations for participant's answers for (benefits of adopting myVisit TH application) arranged in descending order. TH: telehealth

	Using myVisit TH application is beneficial to my patient care and management	myVisit TH application helps save time	Videoconferencing is beneficial	myVisit TH application can improve the quality of care services	myVisit TH application helps save money	Easy use of network	General mean
Mean	4.61	4.52	4.33	4.30	4.11	4.07	4.32
Std. Deviation	.627	.574	.847	.838	.984	.988	0.809
Number of cases	54	54	54	54	54	54	
Order	1	2	3	4	5	6	
Sum	249	244	234	232	222	220	

The mean indicates that the participants see that the myVisit application is beneficial for patient care and helps save time. To a lesser extent, the participants consider the myVisit application easy to use through the internet.

SUS scores

With a quick analysis of the numbers for the SUS section, the participants largely answered that most people would learn to use the myVisit application very quickly and that they would use it frequently. Still, at the same time, the use of the myVisit application is very cumbersome and it is unnecessarily complex.

It was also mentioned that the questions of the second part represent SUS questions, and according to studies, a SUS score of more than 68 is considered above average, while anything less than 68 is considered below average; nevertheless, the best approach to evaluate your results is to "normalize" the scores to generate a percentile ranking. Therefore the mean for all SUS question answers is 3.52, equal to 70%, and based on the SUS measurement, the level of satisfaction is above average for families.

## Discussion

Based on study themes, we aim to know the satisfaction of the participants with the use of the myVisit application to communicate with their patients, the first theme discussed the benefits of the possibility of training on the myVisit application and its better availability for a longer period, which allows patients to communicate with their relatives during longer periods, more than 20 participants their challenge was to accept the use of the myVisit application.

The study by Ji Won Shin [13] and others aimed to explore the opinions of families about the VidaTalk application, and the experience of using it to communicate with patients in ICUs, where the participants expressed happiness and gratitude for communicating with the patient, in addition to that the use of the application for communication allowed clear communication and the expansion of the duration of communication, the results of the study were similar to the results of our studies in terms of the importance of the application by allowing direct contact and reassurance on the patient, and the families' feeling of comfort in communicating with their patients.

A study by Birgitta Lindberg [14] and others focused on the use of information and communication technology in home care to communicate between patients, family members, and healthcare professionals, and the results were positive in the use of information and communication technology, as information technology means enhanced self-care for patients in addition to the ease of communication, especially with restricted cases.

Our study agreed with the study of Doucette [15] and others, which showed that the use of modern technology methods such as smartphone applications represents a new and exciting opportunity to enhance communication between patients and their families in critical care units, as the study concluded that the use of these applications helps to reduce communication gaps between families and their patients.

In general, 59% of them have an education level of less than a bachelor’s degree, as the participants expected the presence of a specialized person to receive calls. myVisit application helped part of the participants to follow up on the condition of their patients remotely, especially at the time of the coronavirus pandemic, three of them used the myVisit application more than four times, and this indicates an important experience using the myVisit application due to the number of times myVisit application was used to communicate with patients, “myVisit application helped me check on the patient’s health and daily contact with him through the application.” Six people suggested increasing the time allotted for communication, as they considered that the time is not enough for the family with the patient to check on his health.

The second theme of importance was staying in safe contact with the patient during his recovery period, 33 participants told about the benefits that they obtained during their communication with the patient, as the form of preventing the spread of infection in addition to the possibility of communicating with the patient, especially for families who live outside Riyadh, the most important benefits myVisit application users felt, “myVisit application is important for those who cannot enter the patient, such as a corona patient or far outside the area.” Half of the participants expressed interest in the medical aspect and staying safely away from the patient, in addition to following up on the patient's condition.

Reference back to the SUS section, the answers of the participants according to Table 3 that the general mean of the participants' answers was (4.32), where the paragraph (using myVisit TH application is beneficial to my patient care and management) got the highest mean (4.61), 36 participants choose very agree.

This indicates the importance of the myVisit application and its benefit to the patient. Forty-five participants confirmed that the myVisit application of my visit improves the quality of health care, regardless of other factors, nine out of 45 participants chose Agree and Strongly Agree, and seven participants were neutral in answering this paragraph.

The benefits of using video-streaming communication technology were also useful by 87% of the participants, this indicates the importance of video-streaming communication technology for a large segment of the participants.

We note that the point that cares about participants' desire to use the myVisit application very quickly got the highest results, and this indicates the acceptance of the idea of people using technology remotely, and to what extent it facilitates participants' lives by saving time, effort, and money. When analyzing the answers, we find beyond any doubt that society, in general, accepts the ideas of technology, especially those that facilitate life and is ready to learn them quickly.

## Conclusions

The most outstanding feature of the myVisit application is the facilitation of the patient and the tracking of his health status by his family, especially when the patient is in the ICU and cannot be visited. It also became clear that the myVisit application saves the patient's family time, money, and effort.

One of the most important requests is to increase the time limit for contact with the patient, as many participants complained about the limited time. On the other hand, there is a great interest in technology and its power to change people's lives for the better, especially in the medical field and in cases where things do not go normally, as well as in extraordinary circumstances.

The results show that the system's usability is very good at 71%, indicating a general willingness to use the myVisit application. Participants' interest in the myVisit application and their previous use was also evident by the number of uses, as more than half of the participants used the application three times or more, and one of the most important benefits participants received was reassurance, ease of communication with the patient, and improvement in the patient's health.

There were many requests to hire a person whose role is only to coordinate calls between the family and the patient, with the condition of speaking the Arabic language to facilitate communication. Finally, the overall evaluation of the myVisit application was positive in terms of ideas and content. Some participants were surprised by the existence of such a service that facilitates communication with their patients.

Limitations

The study was conducted and the questionnaire was distributed in King Abdulaziz Medical City, NGHA, Riyadh, Saudi Arabia, where the study was not conducted and the questions were distributed to other medical cities or a city other than Riyadh. Also, one of the other determinants of the study is that it only focused on families who were allowed to use the myVisit app to communicate with their ICU patients were not included. Families who were not allowed to use the app were not included.

## Appendices

Survey

This survey will be used to determine the level of satisfaction and usability of patient-families in the ICU using the telehealth (TH) application myVisit, and it is consisting of three parts, demographic factors, open-ended and rating scale questions, please answer the following question:

Note that: no personal information will be shared, and your identity will remain confidential.

Part One: Demographic Factors

Kindly put a tick next to the appropriate answer

A- Gender:

1. Male

2. Female

B- Age:

1: 18 to less than 34 years old.

2: 34 to less than 44 years old.

3: 44 to less than 54 years old.

4: 54 to less than 64 years old.

5: 64 and over

C- The educational level:

1. High school and less

2: Diploma

3: Bachelor

4: Master

5: PhD

Part Two: Open-Ended Question

1. How many times have you used the myVisit TH application?

2. In your opinion what are the challenges of using the myVisit TH application (you can choose more than one option):

·          Not knowing the use of the myVisit TH application and the benefits of using it remotely.

·          Not having enough time.

·          Adoption of the idea of using the myVisit TH application.

·          Other reason, specify ………………………………………………………..

3. In your opinion what are the benefits of using the myVisit TH application?

4. What are your expectations of using the myVisit TH application?

5. Do you have any comments or suggestions for what improvements could take place in order to enhance the current myVisit application?

Part Three

Please determine the degree of usability of the system by placing a tick in the appropriate place, which is represented in the following options (Table 4):

**Table 4:** Ease of use of myVisit application. TH: telehealth

Benefits of Using myVisit TH application	Strongly Agree (1)	Agree (2)	Neutral (3)	disagree (4)	Strongly disagree (5)
I think that I would like to use this system frequently.					
I found the system unnecessarily complex.					
I thought the system was easy to use.					
I think that I would need the support of a technical person to be able to use this system.					
I found the various functions in this system were well integrated.					
I thought there was too much inconsistency in this system.					
I would imagine that most people would learn to use this system very quickly.					
I found the system very cumbersome to use.					
I felt very confident using the system.					
I needed to learn a lot of things before I could get going with this system.					

Part Four

Please determine the degree of adoption of the system by placing a tick in the appropriate place, which is represented in the following options (Table 5):

**Table 5:** Degree of adoption of myVisit application. TH: telehealth

Benefits of Adopting myVisit TH application	Strongly Agree (1)	Agree (2)	Neutral (3)	disagree (4)	Strongly disagree (5)
Using myVisit TH application is beneficial to my patient care and management.					
myVisit TH application helps save time.					
myVisit TH application helps save money.					
Easy use of network.					
myVisit TH application can improve the quality of care services.					
Videoconferencing is beneficial.					
